# New York Pathological Society

**Published:** 1860-01

**Authors:** E. Lee Jones


### ART. II.—*New York Pathological Society.*

Regular Meeting, Nov. 9, 1859. John C. Dalton, Jr., M. D., President, in the Chair.

(Reported for the Journal by E. Lee Jones, M. D., Secretary.)

### PERICARDITIS—BRIGHT'S DISEASE.

Dr. McCready presented two specimens removed from the same person, a *heart and kidney.*

The gentleman, said he, from whom they were taken, sent for him on the 22d of last month (Oct.). He was well built, fifty-one years of age, and had been always a free liver. I had seen him in the previous spring, when he suffered from a dry cough, which he had for several months before, complaining of general weakness, was somewhat emaciated, and had had, a short time previously, a hemorrhage from the lungs. A physical examination of the chest detected no disease. He was treated in the usual way, and soon felt better. He spent the following summer at Saratoga and lived in his usual way. On his return home, he told him that a couple of weeks previous to that time he had suffered from an attack of gout (which disease, by the by, he was subject to), had treated himself with colchicum (as was his wont), and recovered. Having lost his appetite at that time, he noticed that it had not yet returned, he was unable to retain food upon his stomach—complained, too, very bitterly, that his sexual desires were all extinct. This state of things he had noticed for two or three months.

On questioning him, he found out that he had suffered from irritation of the bladder for a number of years, being compelled to rise three or four times during the night to urinate. He obtained a specimen of his urine for examination, and found that it was light-colored (the quantity passed at one time being quite copious) that it contained a moderate amount of albumen, but no sediment existed. There did not seem to be any œdema about the patient. His countenance being a little pallid, he was accordingly put upon the use of iron and tonics, but without benefit. He seemed, instead, to get more and more restless, his stomach more and more irritable, and withal, he experienced a good deal of difficulty in breathing. Dr. Metcalfe was then called in consultation, but he did not succeed in finding any trouble with the heart or lungs. A few days after, however, a fine crackle was noticed on either side of the chest, extending some little distance below the clavicle, but absent posteriorly. This sign continued for several days, when it was ascertained that there was some slight dullness over the upper part of one scapula, in the supra-spinal fossa, and also bronchial respiration. This again declined, and seemed to disappear, when there was detected a double friction sound over the heart; this increased and became exceedingly loud, so that it was heard distinctly over the whole cardiac region. In the meanwhile, the stomach became more and more irritable, so much so that he could not even retain the stimulants. About this time he suffered from epistaxis, which was considerable, but which was kept up to the day of his death by constantly picking his nose. The last day or two of his life he had some suppression of urine, passing it only once in twenty-four or thirty-six hours. He died yesterday, at three o'clock in the afternoon.

*Post-mortem Examination.*—As was expected to be the case, there were found two or three tubercles at the top of each lung, and the remains of partial pneumonia in the right lung. His heart presented a fine specimen of intense pericarditis, and the kidneys were in an advanced stage of Bright's disease, and presented the appearances described by Mr. Todd under the head of *Gouty Kidney.*

Independent of the fact that the particular form of disease of the kidney was readily diagnosticated, from the presence of gout, the large quantity of urine passed, the smallness of the amount of albumen, and in the absence of any dropsical symptoms,—the case was interesting, as affording an example of what we see occasionally, namely, that organic disease proceeding to this extent, presented no symptoms up to three weeks of death. Here he was living very freely, capable of very considerable fatigue, and had no signs of disease but gout.

### ENCYSTED ADIPOSE TUMORS OF THE INTESTINES.

Dr. Alonzo Clark exhibited two little tumors for the sake of explaining their nature, as far as he understood it, and he is not aware that similar tumors have been reported upon by anybody.

This one is upon the small intestine, partly imbedded in the fat that covers it. It does not seem to have any vascular connection, except possibly at its root. It is about the size of a bean, a little more globular, however, but does not seem to have provoked any irritation by its presence.

Here is another of the same character, sent to him by Dr. Burgess, who, in making a post-mortem examination, picked it out of the peritoneal cavity, where it was lying detached. They were both of the same nature, and he supposes that the one attached, had the patient lived long enough, would have become separated as the other, and been free in the cavity of the peritoneum. Their constitution is simple enough, and entirely harmless. There is a cyst about each, that seems to have been the parent of the growth. The contents are mainly fatty matter. In the tumor that was attached, it was in part adipose material, and in part oily matter. It must have been fluid at the ordinary temperature of body. The adipose cells are distinctly visible, and there is also present a moderate quantity of crystallized fat,—the crystals corresponding in size, shape, and form, to those of stearine. There is a certain amount of *debris* of animal matter, which I suspect to have been the cysts of the adipose tissue.

The older one is a little more complex in its composition, but he believes it originally consisted of the same kind of material. It has a cyst like the other; and its contents is in a great degree oily, with crystals of some oily matters, more in the form of cholesterine, and with some of the triple phosphate, which, as is well known, is very common in many old structures. This contains a material that he first supposed was epithelial, thinking that it must have been formed by friction of the intestinal parietal peritoneum, and thus constituting the nucleus, a rolled-up mass of epithelium. On further examination, it was found not to be epithelium, but the remains of a cyst of the adipose tissue. These delicate membranes are fully infiltrated with oil, which no doubt acted as a means of preservation.

They are, then, nothing more than fatty tumors encysted and having undergone such degeneration as is incident to oily matter under such circumstances, and in different degrees according to their age. He has never happened to meet with any investigations on the nature of these tumors; if any of the members present have been more fortunate, he would be gratified to learn.

Dr. C. next exhibited the specimen of a heart with vegetations upon the mitral valve, with a written history, for a candidate.

### NECROSIS.

Dr. A. C. Post presented a small piece of bone removed from the tibia. It was only interesting in relation to its diminutive size, having kept up an irritation for five years.

### VEGETATIONS ON FREE MARGIN OF MITRAL VALVE.

Dr. T. C. Finnell presented the uterus and heart taken from a large, fat woman, aged forty. Some sixteen years ago, she suffered from a very severe attack of rheumatism, and since that time had complained of pain and palpitation about the heart. A week ago last Sunday she fell in labor with her first child. The gentleman in attendance thought proper to apply the forceps. In the evening after the delivery, the mother was very comfortable, and on the eighth day after, she was well enough to sit up in a chair. That evening she went to bed at eight o'clock, and the husband, on returning home, found her dead, the infant lying upon her arms.

At the autopsy on Monday, the cause of death was found to be vegetations upon the free margin of the mitral valve. There was no other disease of the valves present. The pericardium contained about three ounces of fluid.

He merely presented the uterus to show its appearance a week after delivery, and laid open the left ovary to show the corpus luteum.

### STRICTURE, WITH PERINEAL FISTULA—PELVIC ABSCESS—ATROPHY OF LEFT KIDNEY.

Dr. Markoe presented the *bladder* and *kidneys* with the *ureters* attached, which were taken from a patient forty-six years of age, who was operated upon at the New York Hospital, for stricture with perineal fistula. The operation was performed about a month ago. Notwithstanding the history of the patient was very carefully sifted, we could not, said he, elicit from him the existence of any gonorrhœal trouble that was antecedent. When young, the patient had some difficulty with his water-works, attended with stoppage of urine and the passage of gravel. How many and how large they were, could not be ascertained. From that period he was never free from considerable irritation about the passages. As he grew older (his habits of life being regular) the symptoms began to partake of the character of stricture, so that he would frequently have his water entirely stopped for a time. The urgency of these symptoms, however, would pass off, and an instrument was not required more than once to relieve him. He went on nine years without suffering very severely from the stricture, until about a year before he presented himself to us. At that time abscesses formed in the perineum, which broke and formed one or two fistulæ.

This rendered his life so exceedingly uncomfortable that he was induced to apply for relief. On examination, he was found to be a man in tolerable health, and there was no evidence elicited of any diseased condition of the urinary apparatus, either from examination of the urine, or the general symptoms, which led us to suspect the existence of gonorrhœa.

He was operated upon in the usual way: an instrument of very small size passed through a stricture about one and three fourths or two inches from the meatus, but was completely arrested in the membranous portion.

There was nothing peculiar in the operation itself; it was conducted to its termination without violence, and was apparently as successful as these cases ever are. For eight days after the operation, the patient was perfectly comfortable. The instrument was removed at the end of the fourth day, and from that time it was introduced once or twice in the twenty-four hours. Every thing promised well; when, at the end of the eighth day after the operation, the patient was attacked with chills, attended with cold sweats and tenderness about the wound. The symptoms progressed with extreme rapidity, the chills being markedly irregular; a low form of ataxic fever showed itself, with muttering delirium, and the patient died at the end of four days.

*Autopsy.*—The post-mortem examination detected the existence of a pelvic abscess about the neck of the bladder. This abscess opened into the cut formed by the operation, extended behind the body of the pubis, its walls covered with detritus that looked almost sloughy. The bladder appeared, as far as we could judge, to be perfectly healthy, except being morbidly thickened and somewhat contracted; a circumstance explained by having a stricture so long.

The right kidney was exceedingly enlarged, somewhat congested, but healthy in its structure, weighing 12 oz. The ureter of that side was dilated proportionately to the size of the kidney.

The left kidney was found to have undergone a peculiar change; this feature invested it with great pathological interest. There was no left kidney, but in its place a regular bean-shaped body, two and a half inches broad, little over an inch wide, and an inch thick, which, on being felt, was found to be a bony shell or cast, containing within it some syrupy, reddish-looking matter. The bony casement was nearly complete, the deficiencies being made up by tough membranes. The casing occupied about three fourths of the kidney substance, the rest being formed by fatty tissues.

It is very clearly a case of complete atrophy of the kidney, the original capsule becoming the seat of calcareous degeneration, the tissues within having been absorbed, and leaving the space to be occupied by the dirty fluid matter. This matter contained a large quantity of cholesterine in crystals. The ureter connected with this kidney was entirely obliterated.

This circumstance, and the degree to which the degeneration has reached, seem to point to a very advanced condition of atrophy. It is an interesting question in looking back, to ascertain whether this condition of things, this action terminating in atrophy, may not have commenced in calcareous pyelitis, and only been fully completed at the time of his death, thirty-one years after. The bony character of the covering can be readily appreciated by the touch and sound.

### CHRONIC, SCROFULOUS EPIDIDYMITIS.

Dr. M. presented also the specimen of a testicle, which was removed yesterday at the New York Hospital, and which presented some points of interest. The man said he was in the neighborhood of 58 years of age; in whose history, on carefully studying it, one could find no evidence of constitutional syphilis (though he had had chancre and bubo, was treated by mercury, and, as far as was known to the contrary, recovered). He did not have any of the clear marks of scrofula in early life. At the time of admission, he had, on the right side, an enlarged testicle, which he stated had existed between two and three years, and had been during that time gradually increasing in size. He did not seem to experience much inconvenience, suffering only occasionally from attacks of pain. Some weeks before he came to the hospital, an ulcer about the size of a twenty-five cent piece, with prominent granulations and inverted edges, made its appearance on the scrotum. At first sight it appeared like a fungus; but Dr. M. was disposed to diagnosticate that it was not such, by the slight degree of protrusion which was manifested when the ulcer occupied such a large surface. Whenever we find that extent of space ulcerated, the process of protrusion is exceedingly rapid. This ulcer had remained in this flattened condition for several weeks; and from that circumstance mainly he diagnosticated the disease not to be connected with the testicle substance. On carefully examining the testicle, it was pretty evident that the trouble was in the epididymis, notwithstanding the ulcer was situated over the body of the testicle. He had no hesitation in stating positively that it was a case of chronic, scrofulous epididymitis. Remedies were employed for a considerable period, but with no benefit. The patient being anxious to have the tumor removed, the operation was accordingly performed yesterday.

On examination, it will be seen that the testicle in this enlarged mass is perfectly sound. Careful dissection from behind forward towards the apparent fungus, shows the tunica albuginea to be perfectly healthy, proving that the ulcer was scrotal and not testicular. There was a little hydrocele at one or two points. When we come to the epidydimis, we find it very much thickened—three times its natural size, and studded with abscesses from the size of a pea to a hickory-nut. Several of these contain thick pus, and others exudation-matters. The whole epidydymis was so thickened and disorganized by the products of inflammation, that it was impossible to say on superficial examination, whether these deposits were in the epididymis or about and around it.

Whether this was truly tubercular softening, or whether it was merely the results of a plastic inflammation, not properly tubercular, he is not prepared to say, as the material has not been examined through a microscope.

### URIC ACID CALCULI IN AN INFANT.

Dr. Bibbins stated that he presented, at the last meeting, two of seven uric ucid calculi, passed by a boy, five months old. [See Report last meeting.] Since that time, the child passed six more; five of these he passed without assistance, but the last became impacted, and had to be removed by mechanical means. The urine is slightly acid, and the child has the same symptoms of dysuria as formerly. There are probably more calculi to come, although the condition of the urine would not favor the idea that they are in the forming state. He had made, said he, considerable inquiry, if any had met with uric acid calculi in so young a child; the attending surgeons of the Dispensary (Demilt), the house physician, and the visiting physician, never having seen such a case; and for the eight years that he has been connected with the institution (as visiting physician), having seen in that time upwards of 5,000 children, it was the first case of the kind he had ever seen.

The calculi were examined by Professor Dalton, and found to be composed of uric acid.

Dr. McCready had seen six or seven such calculi passed by a child between six and seven months old. Lithic acid deposits, he remarked, were not very uncommon in infants.

Dr. Gouley recollected to have seen it in the infundibulum and pelvis of the kidney of a child one month old.

PERICARDITIS—ENLARGEMENT OF LIVER—DEGENERATION OF KIDNEY.

Dr. Bauer presented the specimen of a heart removed from a German merchant. The patient had been unwell for a considerable length of time, had complained of asthma, showed a bronzed discoloration of the skin, and was at last attacked with dropsy, and died. He changed his physician so often that no one was able to get much of a history from him. The two physicians for whom Dr. B. made the post-mortem, were only in attendance for the two weeks before his death ; and during all that time the patient's condition was so much enfeebled that no satisfactory examination could be made. The bronzed skin, however, created a suspicion that there was something wrong with the supra-renal capsules. Besides the skin, the sclerotic coat and finger-nails were tinged.

From that circumstance, Dr. B. was satisfied that the hue of the skin, etc., was due to icterus peculiarly modified by the difficulties in respiration. The heart was found to be closely adherent to the pericardium. There had been pericarditis a good time previous to his death ; and from the condition of the valves, it could be inferred almost with a certainty, that there had been also present a slight degree of endocarditis. The organ itself was very much increased in size, the ventricles being four or five times larger than natural. The lungs were healthy, with the exception that, at a few spots, emphysema showed itself, which evidently had been produced by laborious coughing.

The liver was enlarged and congested. There was also some degeneration of the kidney. His urine had been prevented from free discharge by some mechanical cause ; and, as a consequence, the ureters were somewhat dilated. The condition of the liver convinced Dr. B. that it was inefficient in its action, in removing the elements of bile from the blood ; and the passive congestion consequent upon the difficulty in respiration, modified the yellow tinge to a bright hue. He remarked that there were a great many cases recorded where this bronzed skin existed and there was no disease of the supra-renal bodies, and *vice versa.*

INCARCERATED HERNIA.

Dr. B. presented a second specimen, of *incarcerated hernia*, removed from the body of a woman who died suddenly. A physician was called in, and the gravity of the symptoms relating to the stomach, inclined him very strongly to the belief that some corrosive poison had been given, or taken with a suicidal purpose. On post-mortem examination, a hernial tumor was discovered in the groin ; with extensive peritonitis throughout the whole abdominal cavity. Only a part of the caliber of the intestine was strangulated, and that was gangrenous. He noticed a peculiarity in the connection with the extent of the constriction at the neck of the sac,—that it was on all sides, instead of being confined to the situation of Gimbernat's ligament.

---

Regular Meeting, Nov. 23, 1859. John C. Dalton, Jr., M.D., President, in the Chair.

WOUND OF FŒTUS IN UTERO.

Dr. T. C. Finnell presented the lower extremity of a fœtus, with an incised wound of the limb just below the knee joint. The following history was given. The mother of this child was assaulted by a man suffering from *delirium tremens*, who stabbed her in the abdomen; and her husband, in attempting to rescue her, likewise suffered from some wounds, which, however, were slight. The woman was placed in bed, and the wound dressed by a medical man in the neighborhood. Within a week labor came on. The labor was natural—lasting only a few hours—the child being still-born. The physician, making his visit the next day, had his attention called to a wound in the leg of the fœtus, which was situated in the upper third of the limb, passing completely through from behind forward, and severing the head of the fibula. The wound was about half an inch in length, and was larger posteriorly than anteriorly.

The point of interest is to determine whether that wound was inflicted while the child was in the uterus or not. There were no signs of extravasation around the wound, notwithstanding that the injury had been inflicted some days before. Recollecting the doubled-up condition of the fœtus *in utero*, it seemed remarkable that only one wound should be inflicted—all the other parts escaping injury. Then, the absence of any blood in the liquor amnii—which fact was noticed particularly by the gentleman in attendance, is also worthy of notice. On inquiring of the husband how the wound was inflicted, he mentioned that the withdrawal of the knife was followed by a gush of water, and it seems that a small quantity of the liquor amnii escaped. The mother stated that she felt no motion of the child since the wound was inflicted.

The points of the case, then, are these: the absence of any great amount of blood externally or internally, or of any severe constitutional symptoms—for the mother entirely recovered. The wound in the mother was situated at the umbilicus, and was about half an inch in length.

Dr. Dalton asked if it was known how deep the wound was?

Dr. Finnell.—It was supposed to be only through the integument. It was not considered safe to introduce a probe.

Dr. Wood did not think it very remarkable that the liquor amnii was not tinged with blood, as he thought it possible for the fibres of the uterus to close up a small wound immediately after its infliction, as was exemplified in wounds of the ventricles of the heart. He had seen two such cases of the latter injury, where there had been very little if any hemorrhage following the withdrawal of the knife; the patients dying subsequently of pericarditis.

Dr. McNulty asked if Dr. F. was satisfied that there was no escape of blood from the external wound.

Dr. Finnell.—The statement of the husband was to the effect that there was nothing but water escaped, which caused him to be greatly surprised. I supposed that there was some hemorrhage from the cutaneous vessels.

Dr. Dalton.—Was the child dead previous to birth?

Dr. Finnell.—It probably died shortly after the injury was inflicted, as at the time of delivery the putrefactive process had advanced considerably, as was proven by the peeling off of the skin.

Dr. Dalton.—The case is certainly a very singular one. If the escape of watery fluid actually took place through the external wound, there would seem very little reason to doubt that the wound in the fœtus was inflicted at the same time.

Dr. McNulty.—Was the delivery a premature one?

Dr. Finnell.—She fell in labor about the eighth month—the wound in the abdomen being inflicted a week before.

Dr. McNulty thought if the uterus had been wounded, labor would have supervened in a much shorter time.

Dr. Briddon asked if it was possible that the limbs of the fœtus could be so completely transfixed, when it was so well known that they were grasped in the operation for version, only with a great deal of difficulty.

Dr. Dalton stated that the liquor amnii, as pregnancy advances, becomes less abundant than formerly. It did not seem to him impossible that such a wound should be inflicted if the limb of the child were pressed up against the uterine wall.

Dr. Finnell, in answer to Dr. McN., stated that it was a head presentation.

Dr. Dalton.—That would bring the lower extremities precisely in the position to be wounded in this way. There seems to be good reason to suppose that the child died at the time of the injury—that the labor was brought on by the subsequent putrefaction.

### FRAGMENT OF NUTSHELL EXPECTORATED.

Dr. Clark presented what he termed a quasi-morbid specimen—the half of a chestnut-shell. It has, said he, upon its inner surface, a little incrustation of calcareous matter; the outer surface is quite natural, with the exception of a small amount of this deposit in the furrows. It was coughed up by a gentleman on Friday last. I saw him four years ago, and the story he gave me at that time was, as nearly as I can recollect, as follows: Two years previous to that time (*i. e.*, six years ago), while eating some chestnuts and cracking them with his teeth, he was suddenly seized with an impulse to sneeze, and took a full inspiration with the chestnut shell in his mouth. Immediately after, he had the conviction that something went down his throat. He began to cough considerably, but after a time he ceased to be oppressed. From that time until I saw him he had been coughing considerably, and raising more or less every day. I was called to examine him with the view to ascertain if he had consumption. I did not find any evidence of tuberculous disease, but I noticed that the breathing in the right lung was not so forcible, and the respiratory murmur was more feeble than in the other, and asking for his antecedents, he related this story. From the time I saw him, perhaps a year after, he was attacked with a severe bronchitis, I tended him for two or three days, and then Dr. Wood was good enough to take charge of him. During that time I listened repeatedly, having the idea of the chestnut-shell in my mind. Having found respiration unequal, and having no probability for it in a diseased deposit, the conviction was strong to my mind that the chestnut-shell was there. I told him so in the first examination. He went through bronchitis as well as most persons do—it being attended with a pretty active febrile excitement. From that time until now, I am not aware that he has had any illness except the cough.

I should remark, that the gentleman's general health was good; he was quite stout, and had rather a florid color; in fact, his appearance, as much as his physical examination, helped to exclude the element of tubercles, in my examination.

On Friday morning, he coughed as usual, three or four times, raising a little mucus, and then with a cough not more violent than the others, raised this (specimen of chestnut-shell), enveloped in semi-transparent or opaque mucus. The shell, when coughed, was about its natural form, but was cut, and partly split open, by being carried in the vest pocket,

The maceration for six years does not seem to have altered it materially, it being only somewhat softened, and having a deposit upon its inner and outer surface.

From Friday until to-day (Wednesday), he has coughed as usual. I suggested to him that there was probably an irritation there, that would remain for some time to come, and that he might be content that he got rid of it when warm weather came on.

LARYNGISMUS FROM UNSUSPECTED ANEURISM OF AORTA.

Dr. Clark next presented a specimen of the larynx; portion of bronchial tubes, and larger division of bronchi; and part of the aorta, on which was an aneurism; and the heart. It was taken to-day, from a man 33 years of age, who was received into Bellevue Hospital ten days before his death. When he came in, the prominent symptom was difficulty of breathing. I examined his lungs with considerable care, as far as I was able, at the back, for I could not in front, for reasons which I shall give directly.

The history was, that he had some symptoms of tuberculous disease, which, within the last two months, gave evidence of being in an advanced state. The physical signs were not very difficult to recognize, in the examination that I made.

The patient further stated that he had had syphilis within a year or two. The conviction that I had was, that he was suffering from laryngismus, produced by a certain amount of irritation, which irritation had its origin either in a tuberculous or syphilitic ulcer, near or in the larynx. I examined him with some care, and excluded one of these. He had the same difficulty in inspiration as in expiration; this convinced me that there was no œdema glottidis.

In this state, the man continued for a period of ten days, sometimes worse and sometimes better.

The history was incomplete, inasmuch as every attempt on his part to speak, brought on a paroxysm of dyspnœa, which seemed to threaten his life.

We never examined him in front, because his easiest position was upon his side, lying a little forward. That position he kept day and night, and could not even be raised in bed without bringing on a paroxysm that seemed to be dangerous. I was convinced that the difficulty was in the larynx, and not knowing but that it might kill him by spasm, I left directions with the house physician that he should be closely watched, and if the symptoms increased so as to threaten his life, to call for the assistance of the surgeons, and have his trachea opened. No exigency occurred that imperatively demanded surgical interference, until yesterday morning about three or four o'clock, when the house physician was called to him by a very unalarming message, that the patient was not breathing as easily as through the day. When he arrived, he found the patient absolutely blue, the breath being nearly out of him. The Dr. found that there was no time to send for assistance; he immediately opened the trachea with the means he had at hand, and put in a quill until he could send to the surgeon. The patient breathed easier, and a proper tube was soon after introduced. He was pretty comfortable for a period of six hours, and then sank, more from the shock of the dyspnœa before the trachea was opened than for any difficulty of breathing that remained. I did not see him again alive, after the operation. It seems pretty clear, however, from the testimony, that his breathing was quite relieved by the opening.

On making the examination to day, I had my mind on the larynx as the seat of the difficulty. The trachea was opened from below upward, leaving the larynx untouched until it could be examined from above, also. I found the larynx itself very free from any important disease; there was no effusion present, and the vocal cords presented the appearance of being strained. There was no ulcer of the trachea or of any other portion of the breathing tubes; but this *aneurism* lay upon the trachea; and over this aneurism we were able to trace both the recurrent branches going to the larynx. It seemed as if the whole difficulty was due, just simply to a chronic spasm that had brought the larynx in such a condition that air was not allowed to pass between the vocal cords with freedom for ten days, at the end of which time the patient became absolutely suffocated, after which an opening in the trachea was made. The aneurism forms on the inferior curve of the aorta, and is a little novel in shape.

There would have been no difficulty in making a diagnosis, if we could only have been able to get him in a position with a view to examine the heart; such an attempt was indeed made, but one of the gentlemen standing by remarked that he was fearful the patient might die while I was listening. Percussion would have been the only real evidence of such a state of things, for the reason that during the attack of dyspnœa the noise of air rushing through the rima glottidis was so considerable, that I doubt whether we could have heard any murmur if it had been present.

This is the second time I have had to make this confession here; *the complete oversight of an aneurism that proved fatal by laryngismus.*

Dr. Foster remembered a case which a good many physicians examined, without detecting an aneurism. After a time the patient died, and the dilatation was found to be situated on the arteria innominata.

Dr. Clark. That would not produce spasm of the larynx. We overlook the existence of aneurisms often and often enough; but when our attention is called to them, if they are of sufficient size they are pretty sure to be detected. It is true, also, that no one can make out the existence of the small aneurisms at the base.

Dr. Parker asked Dr. C. what would have been the physical signs in the case?

Dr. Clark answered: Pulsation, dullness from the base of the heart up to the clavicle, because it is a little to the left of the subclavian, and dullness reaching over on the left side under the third rib; and I presume there would have been a murmur. The mere fact of dullness and pulsation would have been quite enough under such circumstances.

Dr. Markoe remarked, that laryngismus was not always produced by pressure upon the recurrent laryngeal, but was dependent in some cases upon the sympathetic relations of the breathing tubes, viz., that pressure upon the trachea some distance from the larynx would give rise to that phenomenon. In such cases tracheotomy was attended with a good result.

Dr. Parker remarked, that these patients could not bear to be placed in a straight position. There seems to be a constant tendency, said he, to press the head forward upon the trunk, and if lying upon the back, this position is kept by pillows behind the head. In two cases where I operated, the difficulty was to place the patient's head far enough back. In one of these cases the aneurism was situated on the arch near the innominata, and the right common carotid was entirely obliterated.

Dr. Clark stated, that as to the position of the neck, there was no disposition in this man to carry the head forward; on the contrary he would lie habitually on his side, inclined over upon his face, with his back bent and head drawn back. The position would have been a very convenient one for operation.

### OPERATIONS FOR NEURALGIC AFFECTIONS.

Dr. Parker next presented a specimen of neuromatous tumor, removed from the arm of a young man by Dr. Chas. K. Briddon.

The patient, aged 21 years, said he, I saw some days ago at Dr. Briddon's request; and I asked him if he would be kind enough to bring the patient to the College and operate before the class, as I wished to witness the operation and know something of the case afterwards. It turns out to be a neuroma, situated on one of the large nerves of the arm, which from the symptoms presented seems to be the ulnar. It is about three inches long and of a variable thickness, and the nerve can be seen stretching over its surface and at one point enveloped in the substance of the mass. The tumor was of slow growth, a period of seven years being consumed in attaining its present size. It was examined microscopically by Dr. Wm. H. Draper and found to be identical in composition with those tumors described by Mr. Smith,—fibro-plastic nuclei, cells and fibres, the fibrous element predominating.

Dr. Clark supposed such tumors were, commonly, pretty nearly all made up of fibrous material within the neurolema, the nerve itself being spread out in consequence like a sheath, and its fibres separated to a greater or less extend in proportion to the amount of deposit between them.

Dr. Wood thought that, inasmuch as so small a portion of the nerve was enveloped, the tumor might have been removed and the trunk left entire. Such an operation had been performed by Velpeau.

Dr. Parker remarked, that it would be an interesting fact to ascertain what would in the course of time become of the sensation in the little finger. He had removed a tumor similar to the one presented, from the axilla, and had exsected about 2½ inches from the median trunk of the axillary plexus, which was followed by the loss of power in the thumb and forefinger. The patient was a female about 18 or 19 years of age; and after the lapse of a few months she recovered the power of these fingers sufficiently to knit with ease.

He had also removed about 1½ inches of the posterior tibial nerve from a young man whose great toe was pierced by a darning needle, which broke off, a part remaining imbedded in the flesh. He suffered immensely, and besides had frequent attacks of convulsion. All efforts to remove the foreign body were fruitless; when the operation referred to was performed. The patient was entirely freed from his pain for six months, when he began to have convulsions, and the like, as before. In fact, the attacks of pain were so severe that the only thing to be done was amputation.

Dr. Parker stated, that the practice of exsection of the nerve originated first with the veterinary surgeons, for horses with tender feet.

Dr. Jas. R. Wood stated, that he had divided the branches of the fifth pair time and again, and the pain would return in spite of everything. Even in one of the cases where Meckel's ganglion was removed by Dr. Carnochan for neuralgia, the pain after a time returned with as much vigor as before the operation; showing the tendency for the nerve to grow itself and unite.

Dr. Clark did not think that any deficiency in the nerve matter could be supplied by nerve tissue.

Dr. Peaslee asked Dr. Parker, if the amputation was successful?

Dr. Parker answered in the negative, and remarked that in neuralgia, in regard to the results of section and exsection, he agreed with Dr. Wood. He had cut away the infra-orbital nerve, and even introduced a hot iron wire into the canal, with no other result than staying the progress of the disease for a while.

Dr. Peaslee recollected a case of a patient, who had a very sensitive point on the dorsum of the foot; the pain was indeed so great and persistent, that the anterior tibial nerve was divided with the view of giving him relief. This was the case for six months only; when the pain returning as before, he insisted upon an amputation. Dr. P. upon inquiring into all his symptoms, refused to do it; because he was very sure that the pain was in consequence of injury to the spinal cord itself, and did not believe that any amputation would be of service unless through the small of his back. The patient soon after succeeded in getting some one to amputate his limb, and the Dr. saw him two or three days after the operation, when he reported himself perfectly free from pain. He (the patient) remained in this condition until the flap healed over, perhaps a month or more, when the pain returned, and has been as severe as ever from that time to this. He always referred the pain to the same part of the foot. This went to show that the disease had its origin in that portion of the spinal column from which the nerve tubes which supplied this part originated.

In relation to the reproduction of nerve tissue Dr. P. stated, that Kölliker mentions instances where an inch of nerve tissue was reproduced after it was exsected. Dr. P. supposed, that unless the nerve should be traced to the very centre that it would grow itself; and further remarked, that such a state of things was just as apt to be, as in those cases where bone had been known to have been reproduced without even the existence of periosteum. The length of time required for the nerve to reproduce the portion removed, depends upon the extent of the exsection.

Dr. Dalton suggested that in the case referred to by Dr. Parker, where the nerve was burnt out with a hot iron, it would be interesting to ascertain whether *sensation* returned in the face as before. If you cut off, said he, a nerve and stop the neuralgia for a time, and then the neuralgia returns again, it does not show that the nervous communication is re-established, unless the normal sensations return the same as before. If you have neuralgia of the face, in which the second branch of the fifth pair is affected, and you divide the nerve on the outside of the infra-orbital foramen, the pain stops for two or three months, and then returns; but if the *sensibility* is destroyed permanently, the pain which returns can depend only upon some disease of the nerve inside of the infra-orbital foramen.

The question which Dr. Peaslee raised is important. I believe, that the way in which the return of neuralgia is generally explained is, that the pain depends upon some disease or injury of the nerve, between the point at which the pain is felt and the nervous centres. In that case, of course, cutting off the nerve would not permanently cure the neuralgia. And that seems to be the simplest way of explaining the difficulty where amputation of the extremities is resorted to. You have neuralgia of the foot: you take out the nerve and the neuralgia returns; and notwithstanding you cut off the leg, the pain only ceases for a while. The simplest explanation seems to to be, then, that the pain depends upon some injury of the spinal cord.

It seems to me that there is another explanation, and one that will also apply to neuralgia of the face. You make a simple division of the infraorbital nerve; the neuralgia is arrested for a month, and then returns. At the second operation, the precaution is taken to make half a dozen cuts; the pain ceases for two months. Next, you run a hot needle into the canal; the patient has a longer interval of ease; then another surgeon goes back to the ganglion of Meckel, and there is no neuralgia for perhaps six months; but it still returns. Now, if the pain depends upon some affection of the fifth pair near the Casserian ganglion, or some affection of the brain itself, I don't see why the neuralgia was relieved by the operation at all. It is easy to see why the relief should not be permanent; but why is it even temporary? I believe an operation always does cure temporarily. It appears to me that the disease may be in the fibres of the nerve, say between the infra-orbital foramen and integument. In that case cutting off the nerve at that point would affect a cure permanently, if the disease had not a tendency to travel from the periphery to the center. After a certain time it will affect the nerve inside of the wound. If the same affection, which originally produced the difficulty in the nervous fibres outside of the foramen, creeps nearer and nearer the brain, of course the pain will reappear. This seems to me the only explanation compatible with the fact, that an operation always relieves the pain for a time.

Dr. CLARK, in that connection stated, that in one case Dr. Mott performed section of the nerve thirty-six times, nearly in the same place, and each time the pain returned; the period of release being shortened in proportion to the number of operations performed.

Dr. DALTON stated that such a case as that could be explained by supposing that the nerves unite. In cutting a nerve, you have paralysis as the result, but soon both motion and sensibility will return; so far as that goes, the previous explanation would be entirely sufficient, but there are other cases in which it is not sufficient: that is, for example, when a considerable portion is taken out, a greater part of the nerve than we are in the habit of supposing to be reproduced.

Dr. PEASLEE stated, that in some instances, as in the first operation of Dr. Carnochan, there could be no doubt that the nerve was diseased in that part. He could not explain a case which occurred to him within the last two years, in any other way. A lady had neuralgia in the bulb of the forefinger of the right hand. This had existed for some twelve years, and in the meantime she had resorted to a great many remedies. Both of the digital nerves were divided on each side of the finger; but the pain came again soon after. Finally, she desired that the finger might be amputated, as it was very much in her way, she being unable to touch anything without great pain. The operation was accordingly performed, and the finger removed. It is now two years since, and she has not had a particle of pain since. I can account for this state of things in no other way than, by supposing the disease of the nerve was higher than the site of the first operation.

### SPECIMENS OF DISEASED JAW.

Dr. Parker presented a second specimen, a tumor taken from the under jaw of a man about 28 years of age. The patient was a farmer of good constitution, who called on Dr. Parker, in August last, and presented a tumor connected with his under jaw, which had been growing gradually until a short time before, when it had commenced to increase rapidly. It had existed in all, about seven years. The patient was advised to go into the country until the weather should become cooler. At first, the tumor seemed to form at the roots of the incisors, and have no connection with them; but after a while all these front teeth (incisors) were pushed out, together with one of the cuspidati. The jaw, as the tumor grew, was absorbed in proportion, so that he could bring the molars in contact, the mass at the same time time crowding itself in front, to give the appearance of an hypertrophied tongue, which, at first sight, I supposed it to be. The tongue, however, could be seen when the mouth was wide open, lifting its top over the postero-superior boundary of the tumor. The mass was removed in such a way as to leave a thin shell of bone along the lower edge of the jaw.

The tumor seems to have originated in the outer surface of the bone, from the cellular structure at the bottom of the teeth. On laying it open, it seems to belong to that character of tumor called *Epulis*, or fibroid. When cut into, it had the appearance of boiled lobster-meat. Its outer surface was covered with a mucous membrane, which was ulcerated at one or two points.

Dr. Markoe presented, for Dr. Parker, two specimens of phosphoric disease of the jaw, and remarked that they were interesting in connection with their pathological anatomy, as they differed in a considerable number of points from the pathological anatomy of necrosis, when it occurred under ordinary circumstances. Instead of the separation of the sequestrum from the surrounding parts, we find it surrounded by new bone, a pumice-like material, which adheres to it with so much firmness that it cannot be separated. These two specimens illustrate the fact very beautifully. The history of this case is very short. They were taken from a patient who had labored in a match-factory, during which time he suffered from toothache, and had one of his molars extracted; this was followed by inflammation, suppuration, and necrosis, so that at the end of the fourth or fifth month, a portion of the lower jaw, on the right side, was removed. The wound, after a time, healed up very perfectly, and the patient regained his ordinary state of health; and having left off work for a long time, was tempted to resume it again, when the lower and upper jaw of the other side became also affected. The first specimen shows the pumice-like material covering the whole jaw, there being very little if any evidence of separation between the dead portion and the encasement. This same state of things is noticed in the second specimen.

The unfortunate feature about these cases is, that this pumice-stone exudation represents the reparative attempt on the part of nature to make an involucrum, or rather, it represents what would be accomplished if the dead bone were taken away. In that case, the periosteum would secrete a new bone, which would take the place admirably well of the dead bone. When this pumice-stone exudation takes place, there is no reproduction of the bone. That was the case in this instance; the side of the jaw from which this was removed, had never any bony or fibrinous deposit from the periosteum. Whether the last operation shall be attended with any better result remains to be seen. I should be inclined, however, to prognosticate unfavorably. The patient is now doing exceedingly well.

Dr. Sayre thought that the results obtained by Dr. James R. Wood upon such cases, differed very markedly from those reported by Dr. Markoe in relation to the reproducing power of the periosteum. He recollected one case where both the upper jaws were removed by Dr. Wood (by gradual enucleation), and where a very considerable amount of new bone was deposited in place of the necrosed portion.

Dr. Wood stated that he proposed to bring all his specimens some evening, and compare them with those of Drs. Parker and Markoe, in order to discuss the subject properly.

### FRACTURE COMPLICATED WITH FEVER.

Dr. Sayre presented a specimen of a leg and foot taken from a man twenty-five years of age. The patient had suffered from an attack of typhus fever five years ago, and since then had never enjoyed good health On the 28th of September last he sustained a fracture of the fibula of the left leg, three inches above the ankle, by the falling of a tierce of rice from a cart across his leg. It was thought at the time to be a simple fracture. About forty-eight hours after the accident phlegmonous erysipelas set in, and was soon after followed by typhoid fever. The patient was attended by Dr. Miner, whose untimely death was supposed to have been the result. Dr. Cockroft saw the patient after the death of the Dr., and took charge of the case until to-day, when I saw him for the first time, and assisted the

Dr. to remove the limb. The ankle-joint was found to be entirely destroyed, the articulating surface of the tibia coming in contact with the bare surface of the astragalus, and pus was found to have burrowed between it (astragalus) and os calcis. There were also present four or five openings upon the foot, which freely connected with the cavity of the joint. The lower fragment of the fibula was found to be split in two pieces—the fissure, which was about one or two lines in width, ran down directly into the joint, which caused the synovitis. It was, in fact, a complicated fracture; but there being no displacement recognizable, no diagnosis could be made at the time of the accident.

In connection with the operation he stated that the chloroform was ably administered by Dr. Jones, and remarked that if every one was as careful as he, few if any accidents would happen from its use.

In answer to a question from Dr. Clark, Dr. Sayre stated, as far as he understood (having only seen the case once), about two weeks after the occurrence of the fracture, the symptoms of typhoid fever showed themselves, as manifested by the dry black tongue, sordes upon the teeth and gums, muttering delirium, and small and frequent pulse.

Dr. Clark remarked that the order of events in presenting the case might lead to the supposition that the fever was the typhoid proper, and had its origin in the injury of the foot.

Dr. Sayre was of the opinion that the injury acted as a predisposing cause by lowering the standard of the general system.

Dr. Clark had no idea that a broken foot could give rise to a fever that was communicable. He regarded it as a typhoid condition of the system, sympathetic in character, which was essentially different from that form of typhoid fever described by Louis.

Dr. Parker stated that, at the request of Dr. Cockroft, he saw the case, and was satisfied, from the symptoms that were presented, that it was merely a symptomatic typhoid, and not the fever of Louis. When it was told him that the fever was communicated to Dr. Miner, he unhesitatingly denied the possibility of such a thing; at the same time remarking, that if broken legs were capable of communicating typhoid poison, every surgeon of the city would have had a good dose.

### EXSECTION OF ELBOW-JOINT.

Dr. Wood presented a specimen of the bones of the elbow joint which he resected on Saturday last, from a man about thirty years of age. The patient had come from the western part of the State about three weeks before, and stated that a year previous to this he had trouble about the joint in the way of inflammation:—this continued until such time as *white swelling* resulted. He was very much debilitated in consequence of the drain upon his system, and after getting him in a condition for operation, he was sent to the Hospital on Saturday.

I performed the operation, said he, with two incisions connected by a transverse one, and without any difficulty removed the condyle of the humerus, the head of the radius, the olecranon, and the superior portion of the ulna, so that the whole of the joint was completely exsected. It was dressed in the usual way, and I am happy to say my patient is doing admirably. This is the fourth time I have operated thus. I made in all five efforts; in one, however, the disease extended so high up that I was forced to amputate. Two of these successful cases were in hospital practice, and two in private practice. All have a fair amount of motion in flexion and extension, and also some power of rotation. In all, I have removed pretty much all the condyles, the head of the radius, olecranon, and portion of the ulna.

I am in the habit of going directly in the joint by dividing the olecranon with a large pair of nippers, which gives me a fair opportunity of making my dissection intelligently. It renders the operation very much more easy than by dividing the triceps when just above its insertion in the olecranon. I have seen gentlemen remove this joint by cutting down upon the ulnar nerve, as recommended, and drawing it over the inner condyle, thus preventing it from being injured; but by commencing and dissecting the fibrous tissues about the joint hugging the bone, the nerve was not visible, neither was this the case in three other of my operations. I was gratified to find that I made the section in sound bone, and the prognosis of course is favorable. The exsected portions were found to be very much diseased.

Dr. Sayre remarked, that there was a great improvement in the operation thus performed, inasmuch as dissecting out the olecranon from its fibrous envelope, the attachment of the tendon of the triceps was left remaining; at the same time the periosteum thus left would secrete new bony matter.

